# Potential of DosR and Rpf antigens from *Mycobacterium tuberculosis* to discriminate between latent and active tuberculosis in a tuberculosis endemic population of Medellin Colombia

**DOI:** 10.1186/s12879-017-2929-0

**Published:** 2018-01-08

**Authors:** Leonar Arroyo, Diana Marín, Kees L. M. C. Franken, Tom H. M. Ottenhoff, Luis F. Barrera

**Affiliations:** 1 Grupo de Inmunología Celular e Inmunogenética (GICIG), Albinusdreef 2, 2333 Leiden, ZA Netherlands; 2 Universidad Pontificia Bolivariana (UPB), Albinusdreef 2, 2333 Leiden, ZA Netherlands; 3 Department of Infectious Diseases, Leiden University Medical Centre, Albinusdreef 2, 2333 Leiden, ZA Netherlands; 4 Instituto de Investigaciones Médicas, Facultad de Medicina, Universidad de Antioquia, UdeA, Calle 70 No. 52-21, Medellín, Colombia

**Keywords:** Tuberculosis, Latency, DosR, Rpf, IFNγ biomarkers

## Abstract

**Background:**

Tuberculosis (TB) remains one of the most deadly infectious diseases. One-third to one-fourth of the human population is estimated to be infected with *Mycobacterium tuberculosis* (*Mtb*) without showing clinical symptoms, a condition called latent TB infection (LTBI). Diagnosis of *Mtb* infection is based on the immune response to a mixture of mycobacterial antigens (PPD) or to *Mtb* specific ESAT-6/CFP10 antigens (IGRA), highly expressed during the initial phase of infection. However, the immune response to PPD and IGRA antigens has a low power to discriminate between LTBI and PTB. The T-cell response to a group of so-called latency (DosR-regulon-encoded) and Resuscitation Promoting (Rpf) antigens of *Mtb* has been proved to be significantly higher in LTBI compared to active TB across many populations, suggesting their potential use as biomarkers to differentiate latent from active TB.

**Methods:**

PBMCs from a group LTBI (*n* = 20) and pulmonary TB patients (PTB, *n* = 21) from an endemic community for TB of the city of Medellín, Colombia, were in vitro stimulated for 7 days with DosR- (Rv1737c, Rv2029c, and Rv2628), Rpf- (Rv0867c and Rv2389c), the recombinant fusion protein ESAT-6-CFP10 (E6-C10)-, or PPD-antigen. The induced IFNγ levels detectable in the supernatants of the antigen-stimulated cells were then used to calculate specificity and sensitivity in discriminating LTBI from PTB, using different statistical approaches.

**Results:**

IFNγ production in response to DosR and Rpf antigens was significantly higher in LTBI compared to PTB. ROC curve analyses of IFNγ production allowed differentiation of LTBI from PTB with areas under the curve higher than 0.70. Furthermore, Multiple Correspondence Analysis (MCA) revealed that LTBI is associated with higher levels of IFNγ in response to the different antigens compared to PTB. Analysis based on decision trees showed that the IFNγ levels produced in response to Rv2029c was the leading variable that best-classified disease status. Finally, logistic regression analysis predicted that IFNγ produced by PBMCs in response to E6-C10, Rv2029c, Rv0867c (RpfA) and Rv2389c (RpfA) antigens correlates best with the probability of being latently infected.

**Conclusions:**

The *Mtb* antigens E6-C10, Rv2029c (PfkB), Rv0867c (RpfA) and Rv2389c (RpfA), may be potential candidates to discriminate LTBI from PTB.

**Electronic supplementary material:**

The online version of this article (doi: 10.1186/s12879-017-2929-0) contains supplementary material, which is available to authorized users.

## Background

Tuberculosis (TB) continues to be one of the deadliest infectious diseases. In 2015 WHO reported an increase in the world prevalence and mortality rate of active tuberculosis (TB) disease cases [1], particularly in the developing world. Latent TB infection (LTBI), defined as the absence of clinical symptomatology in the presence of infection, affects an estimated one-third to one-fourth of the human population [2], and as a result of reactivation disease represents the primary source of active TB.

LTBI is traditionally identified as a positive reaction (>5–10 mm induration) in response to the intradermal injection of a protein purified extract of *Mycobacterium tuberculosis* (*Mtb*), named tuberculin | [3]. Although sensitive, the specificity of the intradermal reaction is compromised by previous vaccination with *M. bovis* BCG and/or infection with some non-tuberculous mycobacteria (NTM) [4]. More recently, the Interferon Gamma Release Assays (IGRAs) have contributed to a higher specificity in detection of Mtb infection since these assays utilize proteins encoded in the *Mtb*, but not in the BCG genome, such as ESAT-6 and CFP10 [5–8]. Nevertheless, both assays have demonstrated a low predictive value for progression to the active forms of TB [9, 10].

During the last few years, in vivo and in vitro evidence has indicated that *Mtb* adapts its transcriptional signature to the microenvironmental conditions posed by the host cells, such as macrophages and dendritic cells, and in granulomas of the infected host [11–14]. In addition, these transcriptional changes seem to be necessary for infection establishment [11, 12]. In conditions such as hypoxia, acidic pH, nutrient starvation, and high concentrations of oxygen (ROIs) and nitrogen (RNIs) reactive intermediates and CO_2_ that may all be present inside granulomas [15, 16], *Mtb* requires a group of approximately 50 genes known as the *dosR* regulon [11, 13, 17] to survive and enter dormancy. Indeed, the expression of *dosR* regulon encoded genes has been associated with the non-replicative persistence of *Mtb* [11, 12], showing that some members of this regulon are playing an important role in the maintenance of the latency condition. On the other hand, a family of five genes able to induce resuscitation of dormant bacilli, termed resuscitation-promoting factors (*rpf*), has also been identified in the *Mtb* genome [18]. It has been described that the proteins encoded by the *rpf* genes (*rpf*A-E) are capable of stimulating the mycobacterial growth of non-replicating cells obtained in vitro [18, 19] and play a significant role in the in vivo persistence and reactivation of chronic infection in mice [20, 21]. Additionally, ex vivo studies, have demonstrated that Rpf proteins increased the recovery of *Mtb* from sputum of the patient with active TB [22] and improved the sensitivity of culture-based Mtb test in samples that require long culture times [23].

Given the interest in proteins encoded by the *dosR* regulon and the *rpf* genes, the cellular immune response to some of these members has been studied in different human populations of Africa, Asia, Europe, and America, demonstrating significantly increased responses of LTBI individuals compared to active TB [24–36]. Our studies in a TB endemic community in the city of Medellín, Colombia, have also provided evidence that the *dosR* encoded Rv1737c (NarK, nitrate reductase), Rv2029c (PFKB, phosphofructokinase B), the hypothetical protein Rv2628, and the resuscitation-promoting factors (Rpf), Rv0867c (RpfA) and Rv2389c (RpfD), induced higher production of IFNγ and a higher frequency of T-cells with a CD45RO^+^CD27^+^ (Tcm) phenotype in 7-day stimulation assays of peripheral blood mononuclear cells (PBMCs) of LTBI compared to active TB [29, 30, 33]. Interestingly, this higher response of PBMC from LTBI compared to active TB to Rv1737c, Rv2029c and Rv2628 has also been observed in Africa, Asia, Europe, India and Brazil [24–27, 37], suggesting an immune response independent of the human genetic and environmental background, and possibly of the circulating *Mtb* strains, and thus suggesting the presence of a prevalent immune response to DosR antigens in LTBI.

Given the problems of sensitivity and specificity associated with the immune response to ESAT-6 and CFP10 *Mtb* antigens, the search for immune response biomarkers that more efficiently classify LTBI from active TB is a top priority for the prevalence and incidence of active TB to be reduced. In this study, using variables of the immune response, Receiver Operating Characteristics (ROC curves), CHi-squared Automatic Interaction Detection (CHAID) and logistic regression (LR), we found that the Rv2029c antigen of *Mtb* is a novel classifier of LTBI vs. active TB with high specificity and sensitivity.

## Methods

### Study population

The study population included two groups of individuals: 20 household contacts (HHC) of recently diagnosed (within 2 weeks) pulmonary tuberculosis patients (PTB), and 21 PTB. The HHC were selected from a previous cohort of HHC from PTB, based on their positive response (≥22 pg/ml) to the CFP10 antigen of *Mtb* and the absence of clinical symptoms compatible with clinical TB [38]. The selected HHC have remained healthy for more than 5 years after the incident case diagnostics (long-term LTBI, ltLTBI), and have not received anti-TB treatment accordingly to the Colombian Minister of Health regulations. The active TB status was confirmed microscopically, by detection of acid-fast bacilli (AFB) on sputum smears at the local TB control program’s laboratories, for all TB cases included in the study. The *Mycobacterium bovis* BCG vaccination status was determined based on the identification of the typical scar left after previous vaccination. The blood samples collected from the participants were taken after reading and acceptance of the informed consent forms. This study was approved by the Ethics Committee, Instituto de Investigaciones Médicas, Facultad de Medicina, Universidad de Antioquia (Medellín-Colombia).

### Regents

RPMI-1640 and Dulbecco’s PBS (DPBS) were obtained from GIBCO (Grand Island, NY); Ficoll-Hypaque, and penicillin-streptomycin solution from Bio-Whittaker (Walkersville, MD); dimethyl sulphoxide (DMSO); pooled human serum (PHS) from Invitrogen (Brown Deer, WI; Eugene, OR).

### Mycobacterial antigens

Three *dosR* regulon-encoded (Rv1737c, Rv2029c, Rv2628), two Resuscitation Promoting (Rv0867c and Rv2389c) antigens from *Mycobacterium tuberculosis* (*Mtb*), recognized as immunogenic in a previous study [29], and the RD1 fusion Protein ESAT6-CFP10 (E6-C10) were used in the present study. The recombinant proteins were produced and QC-ed by CLMCF and THMO. The production, quality control, preparation and storage of the antigens was previously described [24, 39]. Briefly, genes were amplified by PCR and cloned by Gateway Technology (Invitrogen, San Diego, CA) in a bacterial expression vector containing an N-terminal histidine tag. The proteins were overexpressed in *Escherichia coli* BL21(DE3) and purified, as described previously [40]. Purity and size were checked by gel electrophoresis and Western blotting with anti-His antibodies and anti-*E. coli* antibodies. Residual endotoxin levels were determined by a Limulus amebocyte lysate assay (Cambrex) and were found to be below 50 IU/mg recombinant protein. Recombinant antigens were freeze-dried and shipped at ambient temperature to the Colombian research site, prepared as described [40] aliquoted and stored at −80 °C until further use. The purified protein extract (RT50) (PPD) from Staten Serum Institute (Copenhagen, Denmark) was also used in this study.

### Isolation of peripheral blood mononuclear cells (PBMCs) and culture conditions

The procedure to obtain and culture conditions for peripheral blood mononuclear cells (PBMCs) were previously described [29, 30, 33]. Briefly, PBMCs were collected from sodium heparin anticoagulated venous blood (10 ml) and separated by Ficoll-Hypaque density gradient centrifugation. PBMC were washed twice in DPBS, counted in a hemocytometer and cell viability determined by trypan blue exclusion (>94% for all experiments). 1.5 × 10^5^ cells/well were seeded in triplicate in 96-well U-bottom plates (Corning Costar Inc., Corning, NY), in a final volume of 200 μl/well of RPMI-1640 supplemented with 100 U/ml of penicillin, 100 μg/ml of streptomycin, and 10% human pooled serum (PHS). Cells were cultured in the presence or absence of 5 μg/ml (final concentration) of PPD, the fusion protein E6-C10, or the selected DosR and Rpf antigens. Cell cultures were incubated at 37 °C, 5% de CO2, and 90% relative humidity for 168 h (7-days). Dead cells were determined by staining with 7AAD (Thermo Fisher Scientific, Carlsbad, CA). The number of viable cells at the end of the culture period was >80% for all experiments.

### IFNγ quantitation

Quantitation of IFNγ present in the supernatants of non-stimulated and antigen-stimulated PBMCs was performed by the Luminex technology using a commercial kit from Millipore (Millipore, Billerica, MA, USA), and a Luminex reader (Bioplex 200 Analyzer, BioRad, Laboratories Inc), as previously reported [29].

### Statistical analysis

The IFNγ levels in response to antigens were expressed as net values. The Shapiro-Wilk test was used to test normality in data distribution. The difference in the IFNγ levels between LTBI and PTB was determined by the non-parametric U-Mann Whitney test. The differences in gender and BCG scar between LTBI and PTB was tested by the Chi-square statistics. The capacity of IFNγ levels to discriminate between LTBI and PTB was tested using Receiver Operating Characteristics (ROC) curves. The cut-off level of IFNγ that determines the highest sensitivity and specificity for each of the antigens was identified by the Youden index (calculated using the formula: sensitivity + specificity - 1) [41]. The cut-off levels were used to categorize the high and low responses to IFNγ to each one of the antigens, and later to determine the antigens predictive capacity for disease status (LTBI and PTB). For this purpose, multivariate approaches were used. The multiple correspondence analysis (MCA) was used to determine the response profiles to each antigen based on disease status while the CHi-squared Automatic Interaction Detection (CHAID) [42], was used to determine the antigens that better influence the prediction of being LTBI or PTB. To quantify the degree of association and the influence of the IFNγ response levels of each antigen on the disease status, a logistic regression model was built using a 25 LTBI and PTB subsample that was further validated with a 16 LTBI and PTB subsample. For model estimation, the Bootstrapping method with 100 repetitions was used including the first subsample and all antigens. For model validation, the coefficients obtained with the first 25 subsample (16 LTBI and 9 PTB) were used, and the calibration was evaluated by using the percentage of correct classification (87.5%), the Kappa statistic (0.75 CI95%: 0.42–1.0), and the AUC (Area Under the Curve, 0,94 (IC95%: 0,83–1,0) with its respective confidence interval. The Forward method was also explored to get information on the top antigens to predict active disease. Statistical analyses were performed using the Statistic Package for Social Science Program (SPSS version 21.0, Chicago, IL, USA). Statistical differences ≤0.05 were considered significant.

## Results

### Demographic data

The median age of the LTBI group was 38.5-years old (IQR = 26.75–52.75), 55% males, and 80% had a visible BCG-scar. The group of individuals with active pulmonary TB (PTB) had a median age of 28-years old (IQR = 24–41), and 71% were BCG-scar positive. No significant differences in age, gender or BCG-scar positivity were observed between both groups of individuals (data not shown).

### Capacity of the individual antigens to discriminate between LTBI and PTB disease status

**Fig. 1:**
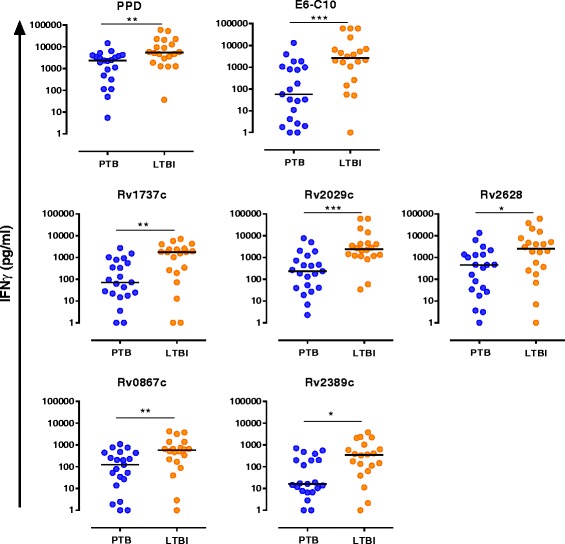
*Comparison of the IFNγ levels in LTBI and PTB in response to RD-1 Esat6-Cfp10 (E6-C10), DosR and Rpf antigens.* PBMCs (1 × 10^5^) were cultured for 7 days, in the presence or absence of PPD, E6-C10 and the DosR and Rpf antigens. IFNγ levels in LTBI (orange circles) and PTB (blue circles) were determined by Luminex. Mann-Whitney U-test was used to calculate statistical differences between groups and *p*-values are shown in the graphs (*, *p* < 0.05; **, *p* < 0.01; ***, *p* < 0.001)

The utilization of RD-1 antigens of *Mtb* in the commercially available IGRAs has contributed to a higher specificity for infection detection in comparison to the tuberculin skin test. Similar to the TST, however, IGRAs cannot discriminate between LTBI and active TB [43–45]. Therefore, the identification of new (host and/or pathogen) biomarkers that discriminate between latent and active TB is a necessity [3]. Thus, we determined the in vitro production of IFNγ by PBMCs from LTBI and PTB in response to selected *Mtb* DosR (Rv1737c, Rv2029c and Rv2628) and Rpf (Rv0867c and Rv2389c) antigens [29, 30, 33] using 7-day PMBC stimulation assay. LTBI individuals displayed significantly higher levels of IFNγ in response to PPD (*p* = 0.004), E6-C10 (*p* < 0.001), Rv1737c (*p* = 0.004), Rv2029c (*p* < 0.001), Rv2628 (*p* = 0.017), RpfA (*p* = 0.009) and RpfD (*p* = 0.013) (Fig. 1; Table 1 and Additional file 1: Table S1).

**Table 1:** Abilities of the IFNγ in response to PPD, E6-C10, DosR and Rpf antigens to discriminate between LTBI and PTB

Antigens	PTB Median (IQR)	HHC-LTBI Median (IQR)	*p*-value*	AUC (95% CI)	Cut-off (pg/ml)	Sensitivity (%)	Specificity (%)
DosR							
Rv1737c	70.7 (18.8–664.2)	1742.0 (209.2–2528.0)	0.0041	0.76 (0.60–0.92)	1014.96	65.0	90.5
Rv2029c	233.4 (38.9–961.3)	2421.0 (1182.0–4426.0)	0.0003	0.82 (0.69–0.96)	763.15	90.0	76.2
Rv2628	452.3 (29.1–1363.0)	2489.0 (281.0–7006.0)	0.0173	0.72 (0.55–0.88)	1569.41	65.0	81.0
Rpf							
Rv0867c	121.8 (19.3–437.2)	576.8 (181.0–1224.0)	0.0091	0.74 (0.58–0.89)	454.89	65.0	81.0
Rv2389c	15.5 (6.3–200.3)	349.5 (74.72–932.6)	0.0126	0.73 (0.57–0.89)	28.36	85.0	61.9
Control							
E6-C10	56.5 (2.4–1027.0)	2623.0 (461.0–6761.0)	0.0006	0.80 (0.66–0.94)	1070.36	75.0	81.0
PPD	2355.0 (401.3–3949.0)	5453.0 (2137.0–18,874.0)	0.0042	0.76 (0.60–0.91)	4464.11	65.0	86.0

*IQR* Interquartile Range. *AUC* Area Under the ROC Curve. *CI* Confidence Interval

^*^p-value for the Mann-Whitney test

Then, ROC curves were used to evaluate the capacity of the antigen specifically induced IFNγ levels to classify LTBI from PTB individuals and to determine the optimal cutoff for each antigen. ROC curves showed that IFNγ production in response to the studied antigens allowed to differentiate LTBI from PTB with areas under the curve (AUC) higher than 0.70; moreover, from the analyses the Rv2029c antigen was found to have the highest contribution to the higher discrimination (Table 1).

### Utility of antigens combinations to discriminate between LTBI and PTB

**Fig. 2:**
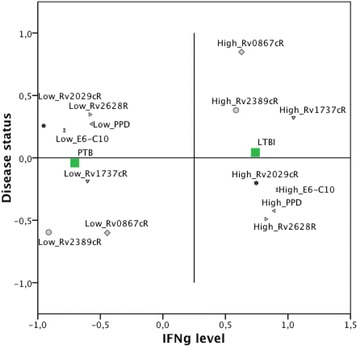
*Multiple correspondence analysis for the IFNγ levels in response to RD-1 Esat6-Cfp10 (E6-C10), DosR and Rpf antigens in LTBI and PTB*

To fully evaluate how the IFNγ response to all antigens could improve the discriminatory capacity between LTBI and PTB, we used multiple correspondence analysis (MCA), a technique for nominal categorical data used to detect and represent underlying structures in a dataset [46]. The MCA results revealed that LTBI individuals are associated with a profile characterized by higher levels of IFNγ in response to the different antigens while PTB are associated with a lower IFNγ profile (Fig. 2).

**Fig. 3:**
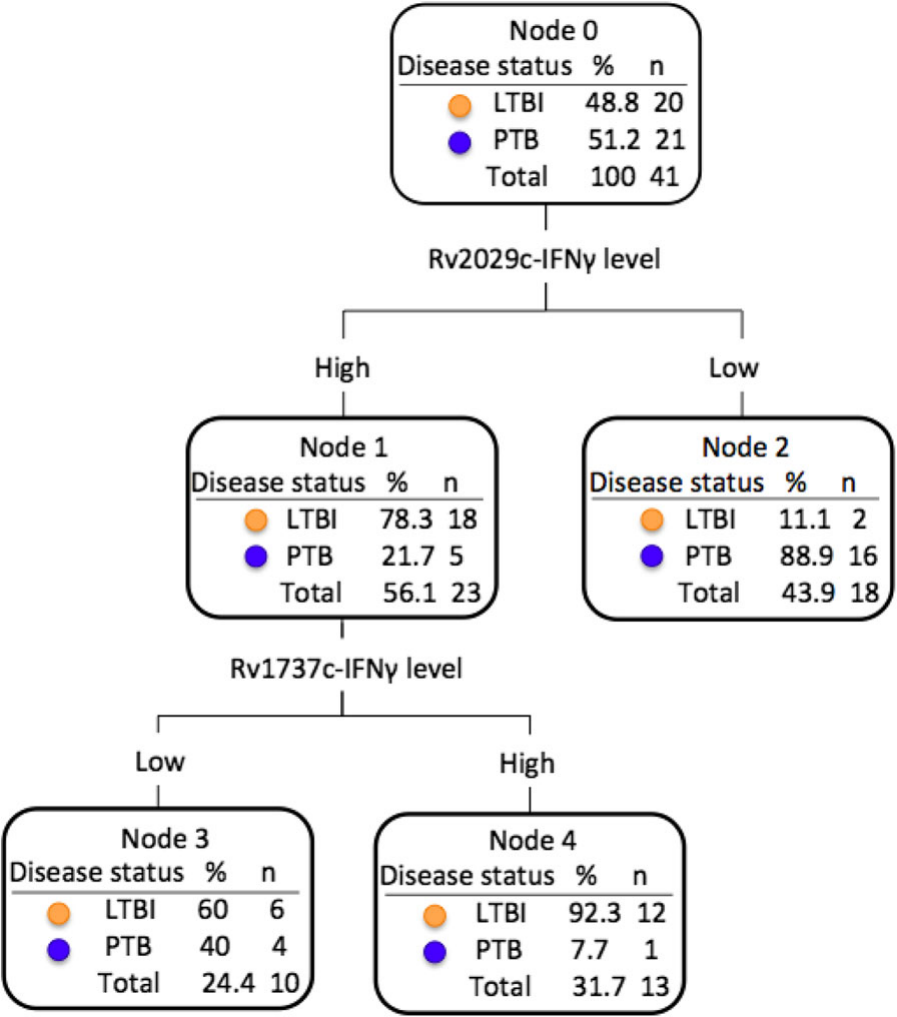
*Decision-tree diagram based on CHAID analysis, showing the antigens that better influence the prediction of being LTBI or PTB*

To identify the variable that better predict the disease status (LTBI or PTB), we performed an analysis based on decision trees (CHAID), to determine which antigens better influence the prediction of being LTBI or PTB. This analysis showed that the higher IFNγ levels produced in response to the DosR antigen Rv2029c (PfkB), is the antigen that best predicted disease status (Fig. 3). The individuals that presented with higher levels of IFNγ in response to Rv2029c display a higher probability of being latently infected (78.3%; Node 1, Fig. 3), while individuals that produced low levels of IFNγ in response to Rv2029c displayed a higher probability of being PTB (88.9%; Node 2, Fig. 3). Also, this method correctly classified 82.9% of the individuals.

To confirm the single antigen that best differentiates between PTB and LTBI, we used the Forward selection method of stepwise regression. The results (data not shown), confirmed Rv2029c as the antigen that best discriminates between those two conditions. Moreover, employing a cross-validation strategy as described in the Statistical Analyses section, this analysis showed that a combination of four antigens E6-C10, Rv2029c, Rv0867c, and Rv2389c, are those that provide the greatest discrimination between LTBI and PTB (Table 2). Using this model, it was possible to construct a prediction rule allowing separation of latent from active TB (Classification score*:* −2,35 + (0,42 x PPD) + (1,90 x E6C10R) + (0,48 x Rv1737cR) + (2,45 x Rv2029cR) + (0,138 x Rv2628R) + (1,86 x Rv0867cR) – (1,87 x Rv2389cR) Probability of LTB: 1(1 + e–classification score)). The validation of this model in a sub-sample (*n* = 16), showed a high capacity to differentiate LTBI from PTB with an AUC = 0,94 (95% CI:0.83–1.0). Additionally, this model correctly classified 85,7% of the LTBI and 88,9% of the PTB.

**Table 2:** Capacity of PPD, E6C10, DosR and Rpf antigens to discriminate between LTBI and PTB

Model						
		B	Bootstrap^a^			
			SE	Sig. (bilateral)	CI (95%)	
					Inferior	Superior
Step 1	PPD	0.424	34.863^b^	0.139^b^	−42.725	76.794
	E6C10	1.899	43.275^b^	0.025^b^	−62.260	119.469
	Rv1737c	0.478	30.405^b^	0.152^b^	−41.714	76.074
	Rv2029c	2.454	40.806^b^	0.038^b^	−39.811	151.793
	Rv2628	0.138	32.905^b^	0.253^b^	−77.272	41.945
	Rv0867c	1.860	35.250^b^	0.025^b^	−36.482	107.89
	Rv2389c	−1.865	39.777^b^	0.025^b^	−132.135	73.181
	Constant	−2.354	31.571^b^	0.127^b^	−95.683	−0.056

^a^Boostrap results based on 100 bootstrap samples, ^b^Based on 78 samples; *SE* Standard Error, *CI* Confidence Interval, *Sig* Significance

## Discussion

The immune response to PPD and IGRA antigens has a low power to discriminate between LTBI and PTB [44, 45]. Indeed, the predictive value of IGRAs for progression to TB disease is low and slightly but not significantly higher than that of the TST [9, 45]. Therefore, new biomarkers are urgently needed to facilitate the diagnosis of LTBI [3].

Herein we have evaluated the potential of the *Mtb* antigens E6-C10, the DosR Rv1737c, Rv2029c, and Rv2628, and the Rpf RpfA and RpfD to discriminate latent TB infection from active TB. The DosR antigens evaluated in the present study induced a higher production of IFNγ in stimulated PBMCs from LTBI compared to PTB. Also, the production of IFNγ to DosR antigens showed a high probability to discriminate disease status (AUC >0.70). Different studies, including ours, indicate that LTBI preferentially recognizes DosR antigens compared to PTB in different human populations [24–26, 29, 30, 32, 33, 35, 37]. In this study, Rv2029c (pfkB) was included in the model that better-predicted disease status, with a correct classification of 78.3% of LTBI and 88.9% of PTB, according to the analysis based on decision trees (CHAID). Additionally, the logistic regression analysis using the forward method also showed Rv2029c as the antigen that better discriminates between those two conditions (data not shown).

Recent studies have highlighted the immunological importance to Rv2029c. Vaccination of mice with DosR antigens, including Rv2029c induced strong humoral and/or cellular Th1-type (interleukin-2 and gamma interferon) immune responses [47]. In humans, a stronger response to Rv2029c in LTBI compared to PTB has been reported in the Netherlands [24], Africa [25, 35], Japan [26], China [36], Brazil [27], and Colombia [29, 30, 33]. Remarkably, in a one-year longitudinal study in Brazilian subjects recently exposed to TB, classified by IGRA and TST positivity, PBMCs stimulation with latency antigens, including Rv2029c, it was found that combining the IFNγ responders to Rv2029c, Rv2031c plus Rv2034 detected 90.3% of IGRA-RD1(+) and 66.7% of TST(+) contacts, while 95% were identified by classifying them as TST(+) IGRA-RD(+) and 11% as TST(−) IGRA(−). Moreover, in the follow-up, the TST converters also demonstrated an IFNγ conversion to Rv2029c and Rv2031c, whereas the only TB incident case was detected via IGRA-Rv2029c and TST previous to developing TB [25]. In another study, the LTBI diagnostic performance of Rv2029c was higher than Rv2628 and Rv1813c by ROC evaluation [36]. Furthermore, in a study in of the in vitro immune response to the DosR antigens Rv1733c, Rv2029c, Rv2628 before and after 2-week anti-tuberculosis treatment in Ghanaians PTB, it was found that the second week of effective chemotherapy was characterized by a general increase in cytokine response to *Mtb*-specific antigens suggesting improvement in cellular response to therapy [48]. Thus, our studies strengthen the observation that Rv2029c may constitute a relevant biomarker of LTBI. Besides, our findings that Rv2029 was included in the model of logistic regression analysis that best predicted latent and active TB, along with E6-C10 and Rpf antigens, may suggest that Rv2029c is a useful diagnostic candidate who might increase the capacity to discriminate between LTBI and PTB in combination with antigens currently used as such as ESAT-6 and CFP-10, even though much larger studies need to be performed to validate the present results.

Similar to the DosR antigens, the Rpf antigens (RpfA and RpfD) induced a higher production of IFNγ in stimulated PBMCs from LTBI compared to PTB and showed a high probability to discriminate disease status (AUC >0.70). Additionally, these antigens were included in the model that predicted the disease status. The results obtained in this study are consistent with those reported by Chegou and colleagues [49], who found that Rv0867c (RpfA) and Rv2389c (RpfD) were included in antigen combinations discriminating between HHC and PTB. It has been reported that *rpf* genes are differentially expressed at different growth stages, and stress conditions, with *rpfA* and *rpfD*, mainly expressed during early resuscitation [50]. Studies conducted in different human population, including ours, indicate that an immune response to RpfA and RpfD antigens has been preferentially found in LTBI suggesting that the immune response to Rpf antigens may play a protective role against bacilli reactivation [29, 34, 35]. It has been suggested that the bacilli may indeed still be replicating but are controlled by the host immune response during LTBI infection [51, 52]. So, the host may be exposed to antigens from the different metabolic states of *Mtb* in vivo, and the response to these antigens may be detected in in-vitro stimulation assays. Thus, our results suggest that RpfA and RpD antigens also could be added to current diagnostic tests to improve the capacity to discriminate between LTBI and PTB.

On the other hand, and somewhat unexpectedly, our results show that the fusion protein E6-C10 induced a higher production of IFNγ in stimulated PBMCs from LTBI compared to PTB and that the production of IFNγ to E6-C10 antigens showed a high probability to differentiate between LTBI and PTB (AUC >0.70). Although E6-C10 was not selected as the best predictor of the disease status, according to the analysis based on decision trees (CHAID) and the logistic regression analysis using the forward method, E6-C10 was included in the model that better discriminates between LTBI and PTB. This result is consistent with those reported by Chegou and colleagues [49], who found that E6-C10 was included in the most of the antigen combinations discriminating between presence and absence of TB disease. ESAT-6 and CFP-10 antigens are encoded by genes located within the region of difference 1 (RD1) of the *Mtb* genome, a chromosomal segment absent in the BCG vaccine strains and most of the NTM [7, 8]. So, the utilization of these antigens in the commercially available IGRAs has contributed to a higher specificity for infection detection in comparison to the tuberculin skin test [44, 45]. The RD-1 antigens are described to be secreted during *Mtb* active replication [6, 53]. Additionally, it has been described that the expression of genes encoding early stage proteins such as ESAT-6 is repressed during the stationary phase of *Mtb* growth in the lungs of chronically infected mice [54], suggesting that they might not be expressed optimally during later stages of *Mtb* infection and likely play a much less dominant role during LTBI than proteins from the DosR regulón or Rpf antigens. Collectively these results suggest that the use the DosR antigen Rv2029c and RpfA and Rpf in T cell assays (IGRAs), in addition to E6-C10, could enhance the ability to differentiate LTBI from TB disease especially in a high-burden setting where a mixture of recent and old infections is commonly found [38].

Our study presents some limitations such as the small sample size which therefore should be extended and validated in a more significant number of participants and different human populations; the use of a 7-day in vitro culture assay rather than a more user friendly assay; the absence of a healthy control group; and the lack of longitudinal evaluation of progression to disease. Although a recent infection is more detectable in a short-term stimulation, it has been argued that long-term stimulation is more sensitive to the detection of LTBI than those with short-term stimulation times particularly in regions of high endemicity in which a mixture of recent and old infections are frequently found [32, 55, 56]. Thus, long-term stimulation may be better to measure central memory T-cell responses [55–57]. By using long-term stimulation conditions, our group has previously reported the enhanced ability to detect Tcm cells (CD45RO^+^CD27^+^) in response to mycobacterial antigens [29, 30, 58, 59]. For that reason, we used long-term cultures to define the LTBI status and compared the immune response between the study groups. As previously pointed out, future works should include a prospective evaluation the antigens and host markers identified in this study, using larger sample size, different geographic settings and using preferably short-term assays to detect effector cell responses. Future studies should also evaluate the antigens and host markers in different study populations as such as children, individuals with immune suppression (e.g., due to HIV coinfection, therapy with TNF-α inhibitors, or due to type 2 diabetes), in TB patients after anti-TB therapy, in patients with extrapulmonary TB, and also in individuals with other lung diseases [49].

## Conclusions

We found that in an endemic community for TB of the city of Medellín, Colombia, human PBMC responses to E6-C10, the *Mtb* DosR antigens Rv2029c (PFKB) and the Rpf antigens, RpfA and RpfD, can discriminate latent from active TB, and may be potential candidates for improved diagnostic tests as well as anti-tuberculous vaccines.

## Additional file

Additional file 1: Table S1. Raw IFNγ levels in PTB and LTBI individuals in response to PPD, RD1 Esat6-Cfp10 (E6-C10), DosR and Rpf antigens. PBMCs (1 × 10^5^) were cultured for seven days, in presence or absence of PPD, E6-C10, and the DosR and Rpf antigens (5 μg/ml). Levels of IFNγ in the supernatants were determined by Luminex. Net values were obtained by subtracting the background values (non-stimulated cells) and are shown in the Table. (DOCX 60 kb)

## Abbreviations

AUC: Area under the curve
BCG: Bacillus Calmette–Guérin
Cfp10: Culture filtrate Protein
CHAID: CHi-squared Automatic Interaction Detection
CI: Confidence intervals
Esat-6: 6 kDa early secretory antigenic target
IFNg: Interferon gamma
IGRA: Interferon gamma release assay
LTBI: Latent tuberculosis infection
MDA: Multiple correspondence analysis
Mtb: Mycobacterium tuberculosis
narK2: nitrate/nitrite transporter NarK2
NTM: Non-tuberculous mycobacterium
PBMCs: Peripheral blood mononuclear cells
pfkB: Phosphofructokinase B
PPD: Purified Protein derivative
PRISMA: Preferred reporting items for systematic reviews and meta-analyses
PTB: Pulmonary tuberculosis
RNIs: Reactive nitrogen intermediates
ROC: Receiver operating curve
ROIs: Reactive oxygen intermediates
Rpf: Resuscitation promoting factor
TB: Tuberculosis
TST: Tuberculin skin test
