# Supplementary material for: Potential of DosR and Rpf antigens from Mycobacterium tuberculosis to discriminate between latent and active tuberculosis in a tuberculosis endemic population of Medellin Colombia
Source: BMC Infect Dis. 2018 Jan 8;18:26. doi: 10.1186/s12879-017-2929-0 (PMC5759254; doi:10.1186/s12879-017-2929-0)
Supplement: Additional file 1: Table S1. — Raw IFNγ levels in PTB and LTBI individuals in response to PPD, RD1 Esat6-Cfp10 (E6-C10), DosR and Rpf antigens. PBMCs (1 × 10^5^) were cultured for seven days, in presence or absence of PPD, E6-C10, and the DosR and Rpf antigens (5 μg/ml). Levels of IFNγ in the supernatants were determined by Luminex. Net values were obtained by subtracting the background values (non-stimulated cells) and are shown in the Table. (DOCX 60 kb) [file 12879_2017_2929_MOESM1_ESM.docx]

|  |  | INFγ levels (pg/ml) | | | | | | |
| --- | --- | --- | --- | --- | --- | --- | --- | --- |
| Individual | **Group** | **PPD** | **E6-C10** | **Rv1737c** | **Rv2029c** | **Rv2628** | **Rv0867c** | **Rv2389c** |
| 80100 | PTB | 113.17 | 0.00 | 0.00 | 128.86 | 0.00 | 0.00 | 0.00 |
| 80200 | PTB | 6355.10 | 3924.13 | 550.51 | 4986.70 | 463.87 | 200.51 | 197.72 |
| 80300 | PTB | 3205.99 | 757.90 | 910.61 | 52.38 | 1369.85 | 772.51 | 202.88 |
| 80400 | PTB | 3494.80 | 27.24 | 0.00 | 432.72 | 2116.52 | 0.00 | 0.00 |
| 81400 | PTB | 14788.23 | 1853.38 | 333.14 | 2014.55 | 13412.15 | 441.16 | 10.66 |
| 81500 | PTB | 3845.48 | 94.55 | 777.79 | 1210.64 | 1356.55 | 755.77 | 397.80 |
| 81800 | PTB | 2933.62 | 31.83 | 1006.07 | 182.40 | 25.35 | 121.77 | 197.08 |
| 81900 | PTB | 1135.25 | 0.77 | 347.67 | 462.46 | 32.88 | 480.96 | 551.84 |
| 82000 | PTB | 113.61 | 0.00 | 93.01 | 17.90 | 16.51 | 205.17 | 15.46 |
| 82100 | PTB | 2354.72 | 31.99 | 42.10 | 412.24 | 79.75 | 77.89 | 18.33 |
| 82200 | PTB | 49.65 | 0.98 | 27.09 | 39.50 | 39.18 | 33.52 | 16.33 |
| 82300 | PTB | 916.89 | 9.94 | 16.57 | 233.36 | 460.47 | 1091.57 | 5.52 |
| 82400 | PTB | 4.50 | 1.62 | 2.55 | 1.26 | 2.10 | .87 | 1.86 |
| 82500 | PTB | 486.18 | 174.90 | 60.63 | 263.19 | 335.09 | 252.21 | 477.05 |
| 80500 | PTB | 2444.34 | 1867.77 | 1515.17 | 711.89 | 1295.48 | 52.04 | 116.41 |
| 80600 | PTB | 316.36 | 56.51 | 21.04 | 38.33 | 2.65 | 1.45 | 5.78 |
| 80700 | PTB | 4276.39 | 799.46 | 13.77 | 25.89 | 1004.97 | 51.18 | 12.69 |
| 80800 | PTB | 2238.59 | 991.78 | 129.19 | 1878.89 | 3122.27 | 227.80 | 9.38 |
| 80900 | PTB | 1957.81 | 3.14 | 70.67 | 5.79 | 160.41 | 12.73 | 13.56 |
| 81000 | PTB | 5174.00 | 13166.90 | 2684.24 | 7667.68 | 6317.55 | 433.20 | 704.01 |
| 81100 | PTB | 4052.49 | 1062.31 | 23.40 | 175.05 | 452.28 | 25.89 | 6.72 |
| 17903 | LTBI | 1255.01 | 255.25 | 70.48 | 1173.72 | 1867.24 | 655.50 | 62.37 |
| 18104 | LTBI | 23194.50 | 23347.30 | 11.89 | 14227.57 | 14918.69 | 1.89 | 1.14 |
| 18112 | LTBI | 1279.58 | 1078.42 | 192.36 | 1157.68 | 568.71 | 218.50 | 145.90 |
| 21701 | LTBI | 4724.41 | 3750.73 | 343.16 | 2792.79 | 4698.87 | 87.68 | 38.39 |
| 23401 | LTBI | 1283.76 | 1694.36 | 2330.76 | 1207.09 | 0.00 | 653.98 | 334.46 |
| 27102 | LTBI | 53057.55 | 1807.78 | 1519.96 | 1432.17 | 368.39 | 502.86 | 404.19 |
| 27301 | LTBI | 59937.54 | 59937.54 | 5713.22 | 59937.54 | 59937.54 | 3185.95 | 2097.54 |
| 29003 | LTBI | 2957.40 | 2152.63 | 259.54 | 2095.23 | 5110.00 | 743.85 | 176.61 |
| 29402 | LTBI | 20836.30 | 6892.62 | 1761.82 | 4506.42 | 37418.24 | 627.71 | 111.76 |
| 32910 | LTBI | 9010.40 | 5273.69 | 2590.82 | 21456.22 | 4067.16 | 168.51 | 133.79 |
| 33208 | LTBI | 12986.78 | 59998.84 | 7111.06 | 4184.50 | 21205.19 | 4243.33 | 382.86 |
| 35103 | LTBI | 5741.68 | 2013.88 | 2339.98 | 4065.41 | 4041.62 | 468.62 | 1032.25 |
| 21102 | LTBI | 4651.83 | 0.00 | 0.00 | 814.42 | 67.63 | 0.00 | 0.00 |
| 22607 | LTBI | 5884.00 | 144.71 | 1916.03 | 32.77 | 251.85 | 663.80 | 584.47 |
| 23002 | LTBI | 22839.27 | 59993.64 | 4022.06 | 59993.64 | 7638.15 | 3731.91 | 2227.37 |
| 26701 | LTBI | 1864.00 | 6366.86 | 4198.17 | 1320.63 | 1988.95 | 1397.34 | 3846.65 |
| 29504 | LTBI | 35.99 | 49.20 | 0.00 | 57.33 | 5.97 | 38.47 | 10.15 |
| 34102 | LTBI | 5164.32 | 4571.51 | 1023.86 | 2631.94 | 2988.12 | 338.34 | 633.62 |
| 35603 | LTBI | 3624.41 | 3093.15 | 1814.62 | 3420.91 | 1768.97 | 1383.54 | 364.53 |
| 41302 | LTBI | 9503.90 | 54.77 | 1721.80 | 2209.43 | 168.42 | 525.95 | 2332.55 |

Table S1. IFNγ levels in PTB and LTBI individuals in response to PPD, E6-C10, DosR and Rpf antigens.

IFNγ levels in PTB and LTBI individuals in response to PPD, RD1 Esat6-Cfp10 (E6-C10), DosR and Rpf antigens. PBMCs (1 x 10^5^) were cultured for 7 days, in presence or absence of PPD, E6-C10, and the DosR and Rpf antigens (5μg/ml). Levels of IFNγ in the supernatants were determined by Luminex. Net values were obtained by substractig the background values (non-stimulated cells) and are showed in the table.
